# Point-of-care determination of the frequency of Rhesus(D)-negative blood types and the uptake of anti(D) immunoglobulin among Rh(D)-negative women in Dadu district, Sindh, Pakistan

**DOI:** 10.1371/journal.pgph.0004395

**Published:** 2025-04-09

**Authors:** Lisa G. Pell, Shabina Ariff, Gul Nawaz Khan, Hana Dampf, Shah Muhammad Jokhio, Alvin Zipursky, Jillian M. Baker, Sidrah Nausheen, Sajid Soofi, Shaun K. Morris

**Affiliations:** 1 Centre of Global Child Health, The Hospital for Sick Children, Toronto, Ontario, Canada; 2 Department of Paediatrics and Child Health, Division of Women and Child Health, Aga Khan University, Karachi, Pakistan; 3 Department of Pediatrics, University of Toronto, Toronto, Ontario, Canada; 4 Unity Health Toronto, St Michael’s Hospital, Toronto, Ontario, Canada; 5 Division of Haematology-Oncology, The Hospital for Sick Children, Toronto, Ontario, Canada; 6 Department of Obstetrics and Gynaeocology, Division of Women and Child Health, Aga Khan University, Karachi, Pakistan; 7 Child Health Evaluative Sciences, Research Institute, The Hospital for Sick Children, Toronto, Ontario, Canada; 8 Division of Infectious Diseases, The Hospital for Sick Children, Toronto, Ontario, Canada; PLOS: Public Library of Science, UNITED STATES OF AMERICA

## Abstract

Rhesus (Rh) disease remains a serious problem in low- and middle-income countries. Rh disease prevention requires early identification and prophylactic treatment of Rh(D)-negative women. We evaluated the feasibility of point-of-care identification of Rh(D)-negative women and timely administration of two doses of anti(D) immunoglobulin by lady health visitors in Dadu district, Sindh, Pakistan. Pregnant women were enrolled at two hospitals and followed until 29 days postpartum. Rh(D)-antigen status was determined using the EldonCard2521 test and all Rh(D)-negative point-of-care test results were attempted to be verified using the conventional test tube agglutination method. Rh(D)-negative women were offered two injections of anti(D) immunoglobulin, one at 28 weeks’ gestation and one within 72 hours of delivery. Knowledge pertaining to Rh disease was assessed among participants at study entry and exit, and in a sample of 30 health care providers. All participants (n=1619) had their blood tested with the EldonCard2521, and 279 (17%) women were found to be Rh(D)-negative; however, the conventional test tube method identified one discordant Rh(D)-antigen result. Among 278 Rh(D)-negative women, 254 (91%) and 268 (96%) received their first and second dose of anti(D) immunoglobulin, respectively. The rates of miscarriage (22.1 per 1,000 pregnancies vs. 4.5 per 1,000 pregnancies), stillbirth (33.8 per 1,000 pregnancies vs. 6.7 per 1,000 pregnancies), and neonatal death (35.0 vs. 16.6 per 1,000 live births) were higher among Rh(D)-negative vs. Rh(D)-positive participants. At study enrolment, there was little knowledge pertaining to Rh disease and its consequences among participants and knowledge also varied greatly among health care providers. The high frequency of maternal Rh(D)-negative blood types, high rates of stillbirth, miscarriage, and neonatal death among Rh(D)-negative women and their newborns, and limited and varied knowledge of Rh disease among pregnant women and health care providers, bolsters the need for a wide-scale Rh disease prevention program in Pakistan.

## Introduction

Rhesus (Rh) hemolytic disease is a preventable disease of the fetus and newborn that is caused by incompatible Rh(D) antigens on the surface of maternal and fetal erythrocytes. Women with an Rh(D)-negative blood group may develop anti(D) antibodies when they are exposed to Rh(D) antigens, a process referred to as alloimmunization. Alloimmunization typically occurs at delivery, but anti(D) antibodies can be produced at any time during pregnancy [1]. Since Rh(D) antibodies do not often appear until months after delivery of the first Rh(D)-positive baby, firstborn Rh(D)-positive babies are not usually affected. Rather, the risk of Rh disease increases with increasing birth order [2,3].

The consequences of alloimmunization may include miscarriage, stillbirth or early neonatal death. Surviving Rh(D)-positive infants may present with anemia, hyperbilirubinemia, and severe hyperbilirubinemia, along with its known sequelae including kernicterus, long-term developmental delay, and death [4]. Treatment for Rh disease varies with disease severity and, in the most severe cases, may require exchange transfusion of the newborn or transfusion of the fetus. These procedures are not always feasible in low- and middle-income countries (LMICs), and are costly and not without complications. Thus, the prevention of Rh disease is of paramount importance.

Alloimmunization occurs in about 12 to 16% of Rh-negative pregnancies, but post-partum administration of anti-D-immunoglobulin reduces the risk to about 1.6 to 1.9% [5]. Prenatal administration of anti-D immunoglobulin reduces the risk of alloimmunization even further, to about 0.5% [6,7]. In general, anti-D immunoprophylaxis should be offered to unsensitized Rh(D)-negative women at 28 weeks’ gestation if the fetal blood type is unknown or Rh(D)-positive, and again 72 hours after delivery if the infant is confirmed to be Rh(D)-positive [8,9].

Despite the existence of effective prevention strategies, globally, 373,300 live births, or 276 babies per 100,000 live births, were estimated to be affected by Rh disease in 2010[10], and more recent estimates are unavailable. The burden of disease is estimated to be greatest in countries with neonatal mortality rates (NMRs) equal to or greater than 15 deaths per 1,000 live births [10,11]. Moreover, an estimated 76% and 87% of women at risk of becoming alloimmunized in India and Pakistan, respectively, did not receive any Rh immunoglobulin [11], a reflection of disparities in antenatal care (ANC) globally.

The first step towards preventing Rh disease is the identification of Rh(D)-negative women, followed by prophylactic treatment. Easy-to-use, portable, and affordable blood type testing technologies can bring Rh(D) testing to rural and hard-to-reach communities in LMICs. Such technologies have been available and produced at large-scale since the 1950s [12]. One such example is the point-of-care EldonCard test, which is based on forward haemagglutination, does not require a cold chain, needs only four drops of capillary blood, and yields a result in approximately two minutes that can be interpreted by the human eye. The sensitivity, specificity, and positive and negative predictor values of the EldonCard have previously been shown to be 100% [13].

Here, the feasibility of point-of-care identification of Rh(D)-negative pregnant women and timely administration of anti(D) immunoglobulin in two secondary health care public facilities, a Tehsil Headquarter Hospital (THQ) and a District Headquarter Hospital (DHQ), in Dadu district, Sindh, Pakistan was assessed. Knowledge regarding Rh disease and its consequences was assessed among pregnant women and lady health visitors at enrolment and study exit and was also assessed among health care providers who were not involved in study activities.

## Materials and methods

### Ethics statement

The research protocol was approved by the Ethics Review Committee (ERC) at Aga Khan University (AKU) (ERC No. 4875-Ped-ERC-17) and the Research Ethics Board (REB) at The Hospital for Sick Children (SickKids) (REB No. 1000057283). To address a lower than anticipated recruitment rate, the protocol was amended to add a second hospital recruitment site in November 2018, approximately five months after the study’s launch. All protocol amendments were approved by the ERC at AKU and the REB at SickKids.

### Study design

This prospective cohort study was implemented in Dadu District, Sindh, Pakistan. The study was registered as an observational cohort study with clinicaltrials.gov (NCT03297671) and updated, as needed, to reflect all protocol amendments.

### Participants and setting

Two types of participants were enrolled in this study: pregnant women and lady health visitors (LHVs). All pregnant women who presented to Tehsil Headquarter Hospital (THQ), Johi, or District Headquarter Hospital (DHQ), Dadu, were screened for eligibility to participate in the study. At the time the study was implemented, THQ Johi had 20 beds, supported a population of approximately 72,000 individuals, and conducted approximately 96 deliveries per month. DHQ Dadu had 188 beds, supported a population of approximately 1 million people, and conducted approximately 334 deliveries per month. Both sites were equipped to provide acute, ambulatory, and inpatient care, including basic radiology, laboratory, and surgical facilities. To be considered eligible, pregnant women had to: be at least 13 weeks gestation based on self-reported first day of last menstrual period (LMP); present to one of the two study hospitals (THQ Johi or DHQ Dadu) for ANC; reside within the study’s catchment area at the time of eligibility screening; intend to deliver their baby and remain in the study catchment area for one month after delivery; and, not have been previously enrolled in the study during a prior pregnancy. Women were not eligible for participation if they reported a prior anaphylactic reaction to an injection of an antibody blood product or any chronic disease related to the heart, liver, kidney, or lungs. To be considered eligible to participate as an LHV, individuals had to: be working full-time as an LHV at THQ Johi or DHQ Dadu; have received verbal permission to participate in the study from their direct supervisor; agree to complete a test on baseline knowledge of Rh disease and its consequences; and agree to attend a one-day training and orientation session. All eligible participants were provided with an overview of the research study, including the study’s purpose, procedures, and the potential risks and benefits of participation, and were given the opportunity to ask questions and to discuss with either their family (pregnant women) or their reporting officer (LHVs) prior to deciding about participation. All enrolled participants provided written informed consent.

### Data collection and surveillance

Attempts were made to follow all participants enrolled during pregnangy and their newborns prospectively from enrollment until 29 days postpartum. All data were collected by trained study team members electronically on tablets using standardized questionnaires that were administered in the form of a structured interview. Information on each participant’s knowledge of the causes, diagnosis, prevention, and potential consequences of Rh disease was collected at two time points: after initial eligibility screening, but before written informed consent was collected (baseline knowledge); and, immediately prior to study exit at the 29-day postnatal age visit. Baseline knowledge data were discarded if a participant declined to participate in the study. Surveys that were designed to assess knowledge about Rh disease were developed such that multiple response options were listed for each question, but these options were not read out loud by data collectors. Instead, data collectors were trained to select response options that were similar to the participant’s response. For example, if a participant responded that Rh disease was a ‘disease of the blood’, the data collector could select ‘blood condition’ from the list of response options. Information on each participant’s reproductive history, demographic, household socioeconomic status, and maternal health status, was collected immediately after enrolment. Data were also collected on delivery characteristics and newborn outcomes, including jaundice, within 72 hours of delivery. Jaundice was assessed through a visual assessment of the newborn’s eyes and skin. Newborn outcome data, including vital status, were also collected at the 29-day postnatal age visit. A convenience sample of approximately 30 health care providers that included physicians, lady health visitors, nurses, lady health workers, and midwives, who were neither employed by the study nor engaged in study activities, were identified, and approached at THQ Johi, DHQ Dadu, and other surrounding health care facilities. Each health care worker was asked to respond to a series of questions to assess their knowledge of Rh disease and its potential consequences. As these health care workers were not enrolled into the study or followed prospectively, and only verbal consent was obtained prior to conducting the survey as a mechanism to confirm their agreement to participate. A formal sample size calculation was not conducted to determine the number of non-study associated health care providers who should participate in the knowledge survey. Rather, the number of health care providers was selected to balance operational feasibility with the generation of a meaningful narrative summary that could inform future work.

After the point-of-care Eldoncard test was administered or refused, all enrolled pregnant women were asked a series of questions to assess their perceptions and acceptability towards the point-of-care test as well as their willingness to pay for the test. Acceptability towards receiving anti(D) immunoglobulin and willingness to pay for anti(D) immunoglobulin injections were assessed immediately after each dose was given or refused. A historical exchange rate from December 30, 2018 (1USD=139.1272 PKR), the midpoint of the study, was used to convert Pakistani Rupees to United States dollars.

Ultrasound examinations were conducted by trained ultrasound technicians. Information on fetal presentation, heart rate, and other relevant information including the presence of congenital abnormalities (i.e., hydrops fetalis) were documented. Gestational age (GA) was derived and compared to GA estimates calculated based on self-reported first date of LMP. In cases where there was a difference of greater than 10 days between GA calculated using LMP and the GA calculated using ultrasound, ultrasound dating was used to determine the anti(D)-immunoglobulin administration schedule and the woman’s estimated delivery date (EDD); otherwise, the GA, EDD, and anti(D)-immunoglobulin administration schedule were based on GA calculated from first date of LMP.

In the event of newborn death or stillbirth, and after a two-to-four-week grievance period, parents were given the choice as to whether a verbal autopsy survey was administered. The survey was developed in accordance with guidelines designed by the World Health Organization [14]. If both parents were present, the verbal autopsy questionnaire was preferentially administered to the mother. Where possible, medical records at THQ Johi or DHQ Dadu were also accessed and assessed alongside verbal autopsies. Verbal autopsy data were used to minimize misclassification of early neonatal deaths as stillbirths, but were not analyzed to assign a cause of death.

### Point-of-care blood test

The point-of-care blood test used in this study was the EldonCard 2521 (EldonBiologicals A/S, Copenhagen, Denmark). EldonCard technology is based on forward haemagglutination, requires four drops of capillary blood per test, and yields an ABO and Rh(D) antigen result in approximately two minutes. Results can be interpreted by eye and/or using a smart-phone camera-based application. The dry format cards have a long shelf life and require no cold chain. A detailed standard operating procedure and pictorial guide on how to use and interpret the EldonCard was provided to each health worker during training and detailed instructions for the card’s use are available from Eldon Biologicals (https://eldoncard.com). In brief, a small drop of water was applied onto each of the four circular fields on the card. The soft skin on the participant’s fingertip was cleaned and punctured with a sterile lancet. A drop of blood was guided onto a plastic Eldon Stick and then transferred to a single reagent circle. New Eldon Sticks were used to transfer a new drop of blood to the remaining three circles. The participant’s finger was bandaged, and they were instructed to seek health care should there be any signs of infection (e.g., redness, discharge, pain, or fever). The results from the card were first interpreted by eye and then using an Android smart-phone application developed by EldonBiologicals A/S to interpret ABO blood group and Rh(D) antigen status.

### Administration of Anti-D Immunoglobulin

Women who were identified to be Rh(D)-negative by conventional tube testing, or in cases where conventional test tube data were not available, by EldonCard, were offered two intramuscular injections of anti(D) immunoglobulin (1500 IU per dose, packaged into a 2ml pre-filled single-use syringe). The injection, sold under the name Rhophylac, was available and licensed in Pakistan and manufactured by CSL Behring in Melbourne, Australia. Verbal consent was collected prior to each injection and injections were only performed at THQ Johi or DHQ Dadu by LHVs who were fully trained and licenced to give intramuscular injections. All participants were observed for 30-minutes after receiving an injection for evidence of an adverse reaction. The first dose of anti(D) immunoglobulin could be administered at any time between 28 weeks’ gestation and delivery; however, where possible, administration of the first dose was attempted during the 28^th^ week of pregnancy. Women who were greater than 28 weeks’ gestation at the time of enrollment and were found to be Rh(D)-negative, were immediately offered their first dose of anti-(D) immunoglobulin at that same study visit. All women who were enrolled prior to their 28^th^ week of pregnancy, and who were found to be Rh(D)-negative, were asked to return to THQ Johi or DHQ Dadu at a later date for administration of their first anti(D) immunoglobulin dose; an appointment card was provided and up to a maximum of four reminder calls were placed to ask women to return to the clinic (once a week for 4 weeks after the 28^th^ week of gestation). Attempts were made to coordinate the visit for the anti(D) immunoglobulin injection with the participant’s ANC visit schedule. Rh(D)-negative women received their second injection of anti(D) immunoglobulin within 72 hours of delivery. If the woman was not reachable within 72 hours of delivery, the second dose of anti(D) immunoglobulin was administered at the earliest possible point of contact, up to a maximum of 29 days after delivery. Two dose administrations of anti(D) immunoglobulin were attempted for each Rh(D)-negative participant.

### Verification of Rh(D)-negative point-of-care blood test results and assessment of maternal anti(D) antibodies

All women who were identified to be Rh(D)-negative via the point-of-care EldonCard blood test were asked to provide a venous blood sample to have their Rh(D) antigen status confirmed using the conventional test tube method, whereby a red cell suspension, prepared in a test tube, was mixed with anti-D reagent, centrifuged, and assessed for agglutination. If discrepancies were identified between the two testing methods, conventional test tube method results were treated as gold standard and dictated whether the participant should receive anti(D) immunoglobulin.

Women who were Rh(D)-negative and had miscarried, delivered a stillbirth, or delivered a live born newborn who died within the first 28 days postnatal age, were asked if they would be willing to provide a venous blood sample to test for the presence of anti(D) antibodies. To allow for a period of bereavement, women were approached at least 2-weeks after their reported event. In each case, women were asked to return to the THQ or DHQ facility at which they were enrolled and a trained medical officer collected a 5-6 ml venous blood sample. Blood samples were shipped to Aga Khan University’s laboratory at Dadu where conventional tube titration methods were used to determine the titer of anti(D) antibodies based on the highest serial dilution that resulted in agglutination. Participants were informed of their test results and were encouraged to report their Rh(D) status and antibody results to health care providers during future pregnancies.

### Statistical analysis

Asset index quintiles were generated using the 2017-2018 Pakistan DHS Survey [15]. Participant and health care worker knowledge at both baseline and study exit was summarized using condensed categories of high understanding, some understanding, and low to no understanding, based on the number of correct and incorrect responses provided at baseline and exit surveys (S1 Table). However, for some questions, knowledge was summarized using condensed categories that explicitly described the number of correct or incorrect responses an individual provided (i.e., knows more than 2 correct methods) (S1 Table). The difference in responses between baseline and study exit were compared with a Stuart-Maxwell test, with p-values adjusted by a Bonferroni correction.

Logistic regression was performed to assess the influence of participant characteristics on maternal baseline knowledge of Rh disease and its consequences. Participants were placed into two groups: those who had some baseline knowledge of Rh disease or correctly identified the appearance or cause of jaundice in infants, and those with no knowledge of Rh disease or jaundice. Predictors of knowledge were selected *a priori* based on potential impact on baseline knowledge. All variables of interest were included in a single multivariable logistic regression model.

### Stata version 17.0 was used for all analyses

#### Inclusivity in Global Health.

Additional information regarding the ethical, cultural, and scientific considerations specific to inclusivity in global research is included in the Supporting Information (S1 Checklist).

## Results

### Participant characteristics

**Fig 1:**
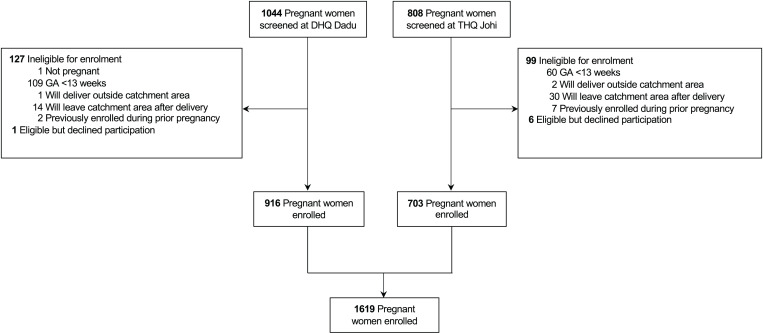
Cohort flow diagram.

Between June 27, 2018 and May 31, 2019, 1852 women were screened for eligibility, 808 from THQ, Johi, and 1044 from DHQ, Dadu. Among women screened, 1626 (88%) were eligible for enrolment, 709 (88%) from THQ, Johi and 917 (88%) from DHQ, Dadu. In total, 1619 pregnant women were enrolled between June 30, 2018 and May 31, 2019, 703 (43%) from THQ, Johi, and 916 (57%) from DHQ, Dadu (Fig 1).

All participant characteristics are summarized in Table 1. Overall, 21% (n=339) of women enrolled in the study were primigravida (Table 1).

**Table 1:** Maternal, demographic, delivery, and newborn characteristics.

Participants, N	1619
Study enrollment hospital, n (%)	
THQ Johi	703 (43)
DHQ Dadu	916 (57)
Maternal age (years), mean (SD)	26.4 (4.7)
Highest level of schooling, n (%)	
No Schooling	1188 (73)
Some Primary	72 (4)
Primary Complete	232 (14)
Secondary or Intermediate Complete	109 (7)
University/Masters Complete	18 (1)
Employment, n (%)	
Not Employed	1296 (80)
Skilled Manual	268 (17)
Unskilled Manual	50 (3)
Other	5 (0.3)
Asset index quintile, n (%)	
Poorest	283 (18)
Poor	751 (46)
Middle	401 (25)
Rich	129 (8)
Richest	55 (3)
Gestational age at enrollment, mean (SD)	24.6 (7.7)
Gravidity, median (IQR)	3 (2,6)
First pregnancy, n (%)	339 (21)
Parity, median (IQR)	3 (2,5)
ANC coverage, n(%)	
1	627 (39)
2	742 (46)
3	192 (12)
4+	58 (4)
Birth outcome, n (%)	
Live Birth	1582 (98)
Stillbirth	18 (1)
Miscarriage	12 (0.7)
Loss of Follow Up^a^	7 (0.4)
Gestational age at delivery^b^, weeks, median (IQR)	40.1 (38.6,41.1)
Preterm gestational age^b^ (<37 weeks), n (%)	196 (12)
Birth facility^c^, n (%)	
Study Facilities	748 (46)
Home	525 (32)
Non-study Private Clinic/Hospital	216 (13)
Other Facility	112 (7)
Birth attendant, n (%)	
Doctor	652 (40)
Nurse/Other Trained	472 (29)
Untrained Traditional/Family	495 (31)
Mode of delivery, n (%)	
Vaginal	1374 (86)
C-Section	227 (14)
Newborn sex^b^, n (%)	
Male	722 (45)
Female	879 (55)
Any sign of jaundice at post-birth exam^d^, n (%)	
None	1440 (91)
Yes	142 (9)
Breastfeeding within 1h of delivery^d^, n (%)	
Yes	1277 (81)
No	304 (19)
^a^Participant Moved, Maternal Death, Maternal Refusal	
^b^Reported for live births and stillbirths	
^c^Birth facility data are missing for 18 participants	
^d^Reported for live births only	

Point-of-care blood test acceptability, interpretation of results and prevalence of Rh(D)-negative antigen among participants

All women enrolled in the study (n=1619) agreed to be tested with the point-of-care blood test. Overall, the point-of-care blood test indicated that 279 (17%) women had an Rh(D)-negative blood type (Table 2). In total, 421 (26%), 471 (29%), 89 (6%), and 638 (39%) participants were found to have A-type, B-type, AB-type, and O-type blood, respectively, according to the point-of-care test. There was perfect concordance between the interpretation of the point-of-care test results by a study worker and the cell phone application (data not shown).

**Table 2:** Prevalence of ABO and Rh(D) antigen blood groups based on point-of-card testing, and administration of prenatal and postnatal anti-D immunoglobulin.

	Blood Group				
	A	B	AB	O	Total
**Rh Positive**	348 (22)	384 (24)	74 (5)	534 (33)	1340 (83)
**Rh Negative**	73 (5)	87 (5)	15 (1)	104 (6)	279 (17)
**Anti-D Immunoglobulin Administration**	**Dose 1: Prenatal administration**		**Dose 2: Post-natal administration**		
Number of anti-D immunoglobulin doses adminstered, n (%)	254 (91)		268 (96)		
Number of missed anti-D immunoglobulin doses, n (%)	24 (9)		10 (4)		
**Reason anti-D immunoglobulin not administered, n (%)**					
Injections not available	3 (13)		0 (0)		
Participant Moved	0 (0)		6 (60)		
Recruitment shortly before birth or missed appointment	2 (8)		0 (0)		
Premature birth or miscarriage	15 (63)		0 (0)		
Maternal death	1 (4)		1 (10)		
Participant family unavailable	1 (4)		0 (0)		
Missing data	2 (8)		3 (30)		

Venous blood samples were collected from 274 (98%) participants who were found to be Rh(D)-negative by the point-of-care test, and conventional blood testing confirmed that 273 (99.6%) of these women were Rh(D)-negative. There was one woman who was determined to be Rh(D)-positive based on conventional blood testing and Rh(D)-negative based on the point-of-care blood test (S2 Table).

Among all 1619 point-of-care tests performed, relatively minor errors in administration procedures were documented during only 5 (0.3%) tests based on direct observation by a study supervisor (S3 Table). Most women (>90%) believed that the point-of-care blood test would improve newborn and maternal health, and that the point-of-care blood test was safe (S4 Table). Similarly, most women (>90%) also reported that their family approved of them taking the point-of-care blood test, that they would recommend the test to others, and that, if needed, they would take the test again in a future pregnancy (S4 Table). Almost all women (>99%) indicated that their preferred location for future point-of-care blood testing would be at either THQ Johi or DHQ Dadu; zero women indicated that their home would be the preferred location of future point-of-care testing (S4 Table).

### Administration of anti-D immunoglobulin

Among the 278 women who had an Rh(D)-negative blood type based on the results of conventional blood testing or, where those data were not available, point-of-care blood testing, 254 (91%) and 268 (96%) received their first (prenatal) and second (post-partum) doses, respectively, of anti-D immunoglobulin (Table 2). In cases where the first dose of anti-D immunoglobulin was not administered, the majority (63%) of doses were missed due to premature delivery or miscarriage taking place prior to when the first dose was to be administered. There were three (13%) instances where the anti-D immunoglobulin injections were not available at the study site by the required time of administration. The majority (60%) of postpartum anti-D immunoglobulin doses that were not administered, were missed due to relocation of participants.

### Pregnancy, birth and neonatal outcomes among enrolled participants

Ultrasound data were collected from 1595 (98.5%) enrolled pregnant women and there were no cases of fetal hydrops observed. Among the 278 Rh(D)-negative women, 56 (20%) were primigravida and 222 (80%) were multigravida. A total of 9 (3.3%) stillbirths and 6 (2.2%) miscarriages were reported among Rh(D)-negative women, compared to 9 (0.7%) stillbirths and 6 (0.4%) miscarriages reported by 1341 Rh(D)-positive women (Table 3). The rates of miscarriage (22.1 per 1,000 pregnancies vs. 4.5 per 1,000 pregnancies, p=0.002), stillbirth (33.8 per 1,000 pregnancies vs. 6.7 per 1,000 pregnancies, p<0.001), and neonatal deaths (35.0 vs. 16.6 per 1,000 live births, p=0.051) were higher among participants who were Rh(D)-negative compared to Rh(D)-positive participants (Table 3). Among live births, signs of jaundice were reported at postnatal exams for 69 (5.2%) infants born to Rh(D)-positive mothers as compared to 73 (28.4%) newborns born to Rh(D)-negative women (Table 3). The NMR was higher among newborns born to Rh(D)-negative multigravida women compared to infants born to Rh(D)-negative primigravida women (44.1 vs. 0.0 per 1,000 live births) (Table 3). Venous blood was collected from 21 (87.5%) of the 24 Rh(D)-negative women for whom stillbirth, miscarriage or neonatal death was reported to assess for the presence of anti(D) antibodies. Of those tested, 8 (38.1%) women were found to be positive for the presence of anti(D) antibodies, 7 of whom were multigravida (Table 3).

**Table 3:** Delivery, neonatal outcomes, and anti-D antibody sensitization, by Rh(D) antigen status and gravidity.

	All participants	Rh(D)-positive participants	Rh(D)-positive participants who were primigravida	Rh(D)-positive participants who were multigravida	Rh (D)-negative participants	Rh(D)-negative participants who were primigravida	Rh(D)-negative participants who were multigravida
Total number of participants, n	1619	1341	283	1058	278	56	222
Delivery outcomes^a^, n (%)							
Live Birth	1582 (98)	1325 (99)	281 (99)	1044 (99)	257 (92)	53 (95)	204 (92)
Stillbirth	18 (1.1)	9 (0.7)	1 (0.4)	8 (0.8)	9 (3.3)	2 (3.6)	7 (3.2)
Miscarriage	12 (0.7)	6 (0.4)	0 (0)	6 (0.6)	6 (2.2)	1 (1.8)	5 (2.3)
Neonatal deaths^b^, n (%)	31 (2.0)	22 (1.7)	6 (2.1)	16 (1.5)	9 (3.5)	0 (0)	9 (4.4)
Any sign of jaundice at post-birth exam, n (%)	142 (9.0)	69 (5.2)	14 (5.0)	55 (5.3)	73 (28.4)	13 (24.5)	60 (29.4)
Miscarriages per 1000 pregnancies^c^	7.4	4.5	0	5.7	22.1	17.9	23.1
Stillbirths per 1000 births^d^	11.3	6.7	3.5	7.6	33.8	36.4	33.2
Neonatal deaths per 1000 live births^e^	19.6	16.6	21.4	15.3	35.0	0	44.1
Participants tested for anti(D) antibodies, n (%)	---	---	---	---	21 (87.5)	3 (100)	18 (85.7)
Miscarriages	---	---	---	---	4 (19.1)	1 (33.3)	3 (16.7)
Stillbirths	---	---	---	---	10 (47.6)	2 (66.7)	8 (44.4)
Neonatal deaths	---	---	---	---	7 (33.3)	0 (0)	7 (38.9)
Negative for Anti-D Antibody	---	---	---	---	13 (61.9)	2 (66.7)	11 (61.1)
Miscarriages	---	---	---	---	4 (30.8)	1 (50.0)	3 (27.3)
Stillbirths	---	---	---	---	5 (38.5)	1 (50.0)	4 (36.4)
Neonatal deaths	---	---	---	---	4 (30.8)	0 (0)	4 (36.4)
Positive for Anti-D Antibody (2 or more dilutions)	---	---	---	---	8 (38.1)	1 (33.3)	7 (38.9)
Miscarriages	---	---	---	---	0 (0)	0 (0)	0 (0)
Stillbirths	---	---	---	---	5 (62.5)	1 (100)	4 (57.1)
Neonatal deaths	---	---	---	---	3 (37.5)	0 (0)	3 (42.9)
Positive for Anti-D Antibody (4 dilutions)	---	---	---	---	2 (25.0)	0 (0)	2 (28.6)
Miscarriages	---	---	---	---	0 (0)	0 (0)	0 (0)
Stillbirths	---	---	---	---	2 (100)	0 (0)	2 (100)
Neonatal deaths	---	---	---	---	0 (0)	0 (0)	0 (0)

^a^Data missing for 7 participants, 1 Rh(D)-positive and 6 Rh(D)-negative, for the following reasons: participant moved, maternal death, maternal refusal.

^b^Vital status at the end of the neonatal period was missing for 2 participants, 1 Rh(D)-positive and 1 Rh(D)-negative.

P-values comparing ^c^miscarriages per 1,000 pregnancies among all Rh positive vs. all Rh negative women was 0.002,

^d^stillbirths per 1,000 births among all Rh positive vs. all Rh negative women was <0.001, and

^e^neonatal deaths per 1,000 live births among all Rh positive vs. all Rh negative women was 0.051. All p-values from Chi-square tests.

### Willingness to pay for point-of-care blood test and anti-D immunoglobulin

In total, 1118 (69%) participants reported that they would have been willing to pay for the point-of-care blood test. On average, the maximum amount participants were willing to pay for the point-of-care blood test was 300.9 ± 70.7 PKR, which was equivalent to approximately $2.16 ± 0.51 USD. By contrast, only 74 (29%) Rh(D)-negative women who received their first dose of RhIg reported that they would have been willing to pay for the injection. Similarly, 91 (34%) Rh(D)-negative women who received their second dose of anti-D immunoglobulin reported that they would have been willing to pay for their second injection. Among participants who were willing to pay for their first or second doses of anti-D immunoglobulin, the maximum median amount that they were willing to pay was 7000 PKR (25^th^, 75^th^: 6000, 9000) and 7000 PKR (25^th^, 75^th^: 6000, 10000), respectively, which was equivalent to approximately $50.31 USD per dose. Most Rh(D)-negative participants indicated that their preferred location to receive future doses of anti-D immunoglobulin was at the THQ or DHQ facility (S5 Table).

### Knowledge related to Rhesus disease

Between enrolment and study exit (139.5 ± 62.9 days), significant increases in knowledge related to Rh disease etiology, consequences, diagnosis, and prevention were reported among study participants (Table 4).

**Table 4:** Participant knowledge about Rh disease and its conseqeunces at enrolment and study exit.

	Enrolment, n (%)	Study Exit, n (%)	p-value
Completed Questionnaire	1619 (100)	1612 (100)	
Had heard of Rh disease	71 (4.4)	1608 (99.8)	<0.001
What is Rh Disease?			<0.001
High Understanding	7 (0.4)	1515 (94)	
Some Understanding	62 (4)	89 (6)	
Low to No Understanding	1 (0.1)	4 (0.2)	
Unknown	1 (0.1)	0 (0)	
Not asked	1548 (96)	4 (0.2)	
Who can get Rh disease?			<0.001
High Understanding	48 (3)	1389 (86)	
Some Understanding	5 (0.3)	5 (0.3)	
Low to No Understanding	13 (0.8)	213 (13)	
Refused	4 (0.2)	0 (0)	
Unknown	1 (0.1)	1 (0.1)	
Not asked	1548 (96)	4 (0.2)	
Why does Rh disease occur?			<0.001
High Understanding	1 (0.1)	499 (31)	
Some Understanding	65 (4)	536 (33)	
Low to No Understanding	1 (0.1)	570 (35)	
Refused	3 (0.2)	0 (0)	
Unknown	1 (0.1)	3 (0.2)	
Not asked	1548 (96)	4 (0.2)	
What happens in severe cases of Rh disease?			<0.001
High Understanding	25 (2)	1560 (96)	
Some Understanding	44 (3)	48 (3)	
Low to No Understanding	0 (0)	0 (0)	
Refused	2 (0.1)	0 (0)	
Not asked	1548 (96)	4 (0.2)	
In whom is it most important to know Rh Status?			<0.001
Mother	53 (3)	1589 (98)	
Baby	5 (0.3)	19 (1)	
Other Family	1 (0.1)	0 (0)	
Refused	6 (0.4)	7 (0.4)	
Unknown	6 (0.4)	0 (0)	
Not asked	1548 (96)	4 (0.2)	
How can you diagnose Rh status of women?			<0.001
Knows >2 methods	4 (0.2)	1375 (85)	
Knows 1-2 methods	62 (4)	175 (11)	
Doesn’t know methods	1 (0.1)	58 (4)	
Refused	4 (0.2)	0 (0)	
Not asked	1548 (96)	4 (0.2)	
How can you prevent Rh disease?			<0.001
Knows >2 methods	3 (0.2)	137 (9)	
Knows 1-2 methods	61 (3.8)	1471 (91)	
Doesn’t know methods	1 (0.1)	0 (0)	
Refused	5 (0.3)	0 (0)	
Unknown	1 (0.1)	0 (0)	
Not asked	1548 (95.6)	4 (0.2)	
Optimal number of prophylaxis doses			<0.001
One	56 (4)	12 (0.7)	
Two	4 (0.2)	1596 (99)	
Three	1 (0.1)	0 (0)	
Refused	4 (0.2)	0 (0)	
Unknown	6 (0.4)	4 (0.2)	
Not asked	1548 (96)	4 (0.2)	
When is the single most important time (in GA weeks) to receive a dose of Rh-Ig if you’re at risk?			<0.001
Both 28w GA & <72h after delivery	2 (0.1)	1515 (94)	
Either 28w GA or <72h after delivery	59 (4)	91 (6)	
Poor to No Understanding	6 (0.4)	2 (0.1)	
Refused	1 (0.1)	0 (0.0)	
Unknown	3 (0.2)	0 (0.0)	
Not asked	1548 (96)	4 (0.2)	
What % of pregnancies are at risk?			<0.001
0% to 5%	1 (0.1)	94 (6)	
6% to 10%	3 (0.2)	928 (58)	
11% to 50%	1 (0.1)	386 (24)	
51% to 100%	1 (0.1)	37 (2)	
Don’t Know	65 (4.0)	163 (10)	
Not asked	1548 (96)	4 (0.2)	
Where did you learn about Rh Disease?			
Own Medical,LHV,Nurse Training	2 (0.1)	17 (1)	
During my pregnancy, source unspecified	12 (0.7)	430 (27)	
Family Member	15 (0.9)	6 (0.4)	
Internet	0 (0)	1 (0.1)	
Doctor	39 (2)	19 (1)	
Nurse	1 (0.1)	88 (6)	
LHV	2 (0.1)	1046 (65)	
LHW/Midwife	0 (0)	13 (0.8)	
Refused	1 (0.1)	0 (0)	
Unknown	1 (0.1)	0 (0)	
Not asked	1551 (96)	16 (1)	
What does jaundice look like?			<0.001
Knows >2 symptoms	86 (5)	850 (53)	
Knows 1-2 symptoms	54 (3)	57 (4)	
Doesn’t know symptoms	16 (1)	703 (43)	
Refused	0 (0)	1 (0.1)	
Not asked	1463 (90)	1 (0.1)	
What causes jaundice?			<0.001
Knows >2 causes	4 (0.2)	800 (49)	
Knows 1-2 causes	14 (1)	709 (44)	
Doesn’t know causes	38 (2)	97 (6)	
Not asked	1563 (97)	6 (0.4)	

Several factors were found to be associated with a woman’s baseline knowledge of Rh disease or jaundice in infants (Table 5). The odds of a woman having any knowledge of Rh disease increased with increasing education level, whether they were employed in skilled manual jobs, and increasing gravidity. Asset index, maternal age, past stillbirths or miscarriages and the number of ANC visits attended did not significantly affect maternal baseline knowledge of Rh disease (Table 5).

**Table 5:** Factors associated with knowledge of Rh disease and its consequences at enrolment.

	Odds ratio	SE	z	P>z	95% CI
Asset Index Quintile (Ref: poorest)					
Poor	0.80	0.19	-0.95	0.34	(0.51, 1.26)
Middle	1.03	0.27	0.11	0.91	(0.62, 1.71)
Rich	0.80	0.29	-0.61	0.54	(0.40, 1.62)
Richer	0.79	0.38	-0.48	0.63	(0.31, 2.03)
Highest Schooling Completed (Ref: No Schooling)					
Some Primary	1.00	0.45	0.01	0.99	(0.42, 2.42)
Primary Complete (Class 5)	2.95	0.63	5.02	0.00	(1.93, 4.49)
Secondary or Intermediate complete (Class 9-11+)	3.87	1.11	4.7	0.00	(2.20, 6.79)
University/Masters Complete	4.76	3.13	2.37	0.02	(1.31, 17.29)
Employment (Ref: Not Employed)					
Skilled Manual	2.33	0.45	4.35	0.00	(1.59, 3.42)
Unskilled Manual	1.27	0.59	0.52	0.61	(0.51, 3.16)
Other	4.31	4.26	1.48	0.14	(0.62, 29.92)
Gravidity	1.12	0.06	2.16	0.03	(1.01, 1.24)
Maternal age (years)	0.99	0.03	-0.36	0.72	(0.94, 1.05)
Past stillbirth or miscarriage	1.17	0.22	0.84	0.40	(0.81, 1.69)
ANC appointments	1.10	0.11	1	0.32	(0.91, 1.34)

SE=Standard Error; CI=Confidence Interval.

Approximately 77% (n=23) of health care providers who were not affiliated with the study and were surveyed self-reported that they had heard of Rh disease; however, true knowledge of disease etiology, consequences, and prevention strategies varied greatly amongst these health care workers (S6 Table). For example, only 6.7% of health care workers were categorized as having a high understanding of why Rh disease occurs and the consequences of severe cases of Rh disease.

## Discussion

With 17% of women found to have a Rh(D)-negative blood type, this cohort of pregnant women in Dadu district, Sindh, Pakistan, has one of the highest reported frequencies of the Rh(D)-negative blood antigen in Pakistan. The frequency of Rh(D)-negative blood types varies markedly across Pakistan with estimates ranging from 5% in Skardu district, Gilgit-Baltistan [16], 5 to 7% in Peshawar, Khyber Pakhtunkhwa province [17], 6 to 8% in Karachi, Sindh province [18,19], and 8% to 20% in Rawalpindi, Islamabad, Safdarabad, Faisalabad, and Gujrat, in Punjab province [20–22]. The genetic determinants of the Rh(D) antigen and its variation across populations are well documented [23]. However, participant recruitment and inclusion criteria varied across the above-mentioned studies and none of them were designed to report population-based prevalence estimates.

In this cohort, the miscarriage, stillbirth, and neonatal mortality rates (NMRs) reported among Rh(D)-negative women were markedly higher than the rates reported for Rh(D)-positive women. While the cause of each miscarriage, stillbirth, and neonatal death cannot be directly linked to Rh disease, the high rates of each of these adverse outcomes among Rh(D) negative women highlights a potential area where efforts could be focused, through enhanced antenatal care and postpartum monitoring programs, to further reduce preventable miscarriages, stillbirths, and neonatal deaths. NMRs among all participants, Rh(D)-positive participants, and Rh(D)-negative participants were 19.6, 16.6 and 35.0 newborn deaths per 1,000 live births, respectively, all of which are higher than the Sustainable Development Goal (SDG) number three global target of 12 deaths per 1,000 live births [24]. As expected, most reports of neonatal death among Rh(D)-negative women occurred in multiparous women, bolstering the need to not only identify every Rh(D)-negative woman, but to identify them early in their reproductive history; the incorporation of blood group screening into routine ANC requirements may improve newborn outcomes.

Recently, the International Federation of Gynecology and Obstetrics (FIGO) and International Confederation of Midwives (ICM) released new guidelines for preventing Rh disease that consider the cost-effectiveness of different dose regimens of anti-D immunoglobulin and prioritizes administration of anti-D by indication [25]. The guidelines indicate that maternal Rh(D) antigen status should be preferentially determined in early pregnancy, as indications for administering anti-D immunoglobulin may arise very early in pregnancy, such as miscarriage or an ectopic pregnancy [25]. However, the guidelines do not explicitly state that maternal Rh(D) antigen status should be preferentially determined during first pregnancies. In this cohort, only 339 (21%) women were primigravida. Among Rh(D) negative women, 222 (80%) were multigravida. Since the likelihood of sensitization increases with each pregnancy, some of the women in this study may have already been sensitized and thus, needlessly received anti(D)-immunoglobulin. Knowledge of whether a woman was already sensitized would be an ideal indicator to guide decision making surrounding the administration of anti-D immunoglobulin; however, in many LMIC settings, particularly in rural areas, this information is not often readily available and nor is it always feasible to measure. Since the sensitization status of all Rh(D)-negative women in this study was not available, nor was it feasible to measure, and the risk associated with administering anti-D immunoglobulin is low, all Rh(D) women received two doses of anti-D immunoglobulin, regardless of sensitization status.

Among participants who indicated that they were willing to pay for the point-of-care blood test, the average maximum amount that they were willing to pay was 300.9 +/- 70.7 PKR, which was equivalent to about 2.16 ± 0.51 USD. The EldonCard retails for approximately 8USD per test, which includes the card, and all supplies needed to administer the test (i.e., sterile lancet, Eldon sticks). Among the small proportion of participants who indicated that they were willing to pay for the first and/or second dose of anti(D) immunoglobulin, the median maximum amount that they were willing to pay was 7000 PKR which was equivalent to approximately $50.31 USD. A single 1500 IU dose of Rophylac retailed for approximately $50USD per dose for the duration of the study. These findings suggest that to successfully scale-up testing for Rh(D) status determination and the administration of anti-D immunoglobulin to Rh(D)-negative women, financial contribution from the government and/or other stakeholders will be required. To reduce costs while still maintaining the effectiveness of an Rh disease prevention program, future programming could reduce the number of doses of anti-D to only a single dose administered within 72 hours of delivery, and to consider using a 500 IU dose instead of a 1500 IU dose, consistent with the FIGO/ICM guidelines [25]. Moreover, pending the generation of more robust evidence on the safety and efficacy of monoclonal anti(D), the cost of future Rh disease prevention programs could be further reduced through the administration of a monoclonal antibody [26]. While this work indicated that the preferred location of future blood testing for women was THQ Johi or DHQ Dadu, and not the participant’s home, it’s important to acknowledge that the participants in this study were facility-recruited and therefore their responses may not reflect the preferences of women who never present to a tertiary level health care facility during pregnancy.

Overall, knowledge about Rh incompatibility and its consequences among pregnant women in this cohort was low, consistent with a prior report of Rh-disease-related knowledge among 350 women in Karachi, of whom 8.3% had a Rh(D)-negative blood group [18]. Knowledge among pregnant women increased markedly in the relatively short time between enrolment and exit from the study, suggesting that the introduction of a brief education and counselling session into ANC visits may significantly increase awareness of Rh disease in this or similar populations. Knowledge about the causes and consequences of Rh disease was also relatively low among the cohort of 30 non-study related health care workers surveyed. Only 77% of health care providers surveyed had heard of Rh disease, highlighting the need for additional education among health care professionals in this region of Pakistan, and exploring Rh disease awareness and education opportunities throughout the country.

A limitation of this study was that, due to budgetary constraints, determination of whether Rh(D)-negative women in the cohort were already sensitized prior to administering anti-D immunoglobulin was not feasible. Thus, as mentioned above, a proportion of the Rh(D)-negative women, especially those who self-identified as multigravida, may have already been sensitized and received Rh(D)-immunoglobulin at no added benefit. Similarly, among Rh(D)-negative women who had their anti(D) titers assessed, we were unable to distinguish between anti(D) due to immunoprophylaxis versus anti(D) that arose from maternal alloimmunization. Second, in three cases, the first dose of anti(D) immunoglobulin was not administered during the first trimester due to a shortage of supplies at the study site. In two of these cases, the women received a dose of anti(D) immunoglobulin within 72 hours of delivery, which is consistent with the high priority guidelines released by FIGO/ICM [25]. However, one of the women migrated outside of the study’s catchment area and could not be reached for her second dose. Third, the exchange rates used to convert PKR to USD were based on values from December 2018, the mid-point of the study. However, fluctuations in currency valuations may impact the amount an individual is willing to pay for the point-of-care test or anti-D immunoglobulin, and should be considered in the design of future programs. Fourth, there was one discrepant Rh(D)-antigen blood type result between conventional tube testing and the point-of-care blood test. While the exact mechanism of the error is unclear, prior research has indicated that Rh(D)-positive individuals may be misclassified as Rh(D)-negative if too much water is applied to the EldonCard [12]. Since confirmatory blood testing was only performed for women who tested Rh(D)-negative using the EldonCard, conclusions cannot be made regarding the misclassification of Rh(D)-negative women as Rh(D)-positive. Fifth, jaundice was assessed visually, which cannot provide an accurate measure of bilirubin. Sixth, due to budgetary constraints, we were only able to assess maternal anti-D immunoglobulin titres in Rh(D)-negative women who experienced miscarriage, stillbirth, or neonatal death, which does not provide a fulsome picture of anti-D sensitization among Rh(D)-negative women in this study. Finally, the study recruited participants in two hospitals in Dadu district, rather than at the community level. By biasing the selection of participants to those who seek ANC at facilities, it’s unclear what the frequency and proportion of Rh(D)-negative blood types or how other outcomes would differ (i.e., knowledge, acceptability, etc.) among women who do not seek ANC or seek care at alternative care facilities.

The high prevalence of Rh(D)-negative blood types, combined with limited knowledge related to Rh(D)-blood typing or the causes and consequences of Rh disease bolsters the need for routine Rh(D)-antigen testing and improved education related to Rh disease among both pregnant women and health care providers in Dadu district, Pakistan. Overall, acceptability of the point-of-care EldonCard blood test by pregnant women and of anti-D immunoglobulin by Rh(D)-negative pregnant women was high, suggesting that the scale-up of a Rh disease prevention program may be feasible from the perspective of the beneficiaries. Future work should consider mechanisms to expand point-of-care blood testing to community settings, and to identify Rh(D)-negative women during their first pregnancy.

## Supporting information

S1 Table Categorizing participant knowledge at enrolment and study exit. (XLSX)

S2 Table Verification of point-of-care Rh(D)-negative antigen results using conventional tube testing. (XLSX)

S3 Table Directly observed protocol compliance during lady health visitor administration of the point-of-care blood test. (XLSX)

S4 Table Acceptability of the point-of-care EldonCard blood test. (XLSX)

S5 Table Willingness to pay for the point-of-care blood test and anti-D immunoglobulin. (XLSX)

S6 Table Knowledge of Rh disease among health care professionals. (XLSX)
